# Comparison of ophthalmic sponges and extraction buffers for quantifying cytokine profiles in tears using Luminex technology

**Published:** 2012-11-16

**Authors:** Aleksandra Inic-Kanada, Andrea Nussbaumer, Jacqueline Montanaro, Sandra Belij, Simone Schlacher, Elisabeth Stein, Nora Bintner, Margarethe Merio, Gerhard J. Zlabinger, Talin Barisani-Asenbauer

**Affiliations:** 1 OCUVAC - Centre of Ocular Inflammation and Infection, Laura Bassi Centres of Expertise, Institute of Specific Prophylaxis and Tropical Medicine, Centre for Pathophysiology, Infectiology and Immunology, Medical University Vienna, Vienna, Austria; 2 Institute of Immunology, Medical University Vienna, Vienna, Austria

## Abstract

**Purpose:**

Evaluating cytokine profiles in tears could shed light on the pathogenesis of various ocular surface diseases. When collecting tears with the methods currently available, it is often not possible to avoid the tear reflex, which may give a different cytokine profile compared to basal tears. More importantly, tear collection with glass capillaries, the most widely used method for taking samples and the best method for avoiding tear reflex, is impractical for remote area field studies because it is tedious and time-consuming for health workers, who cannot collect tears from a large number of patients with this method in one day. Furthermore, this method is uncomfortable for anxious patients and children. Thus, tears are frequently collected using ophthalmic sponges. These sponges have the advantage that they are well tolerated by the patient, especially children, and enable standardization of the tear collection volume. The aim of this study was to compare various ophthalmic sponges and extraction buffers to optimize the tear collection method for field studies for subsequent quantification of cytokines in tears using the Luminex technology.

**Methods:**

Three ophthalmic sponges, Merocel, Pro-ophta, and Weck-Cel, were tested. Sponges were presoaked with 25 cytokines/chemokines of known concentrations and eluted with seven different extraction buffers (EX1–EX7). To assess possible interference in the assay from the sponges, two standard curves were prepared in parallel: 1) cytokines of known concentrations with the extraction buffers and 2) cytokines of known concentrations loaded onto the sponges with the extraction buffers. Subsequently, a clinical assessment of the chosen sponge-buffer combination was performed with tears collected from four healthy subjects using 1) aspiration and 2) sponges. To quantify cytokine/chemokine recovery and the concentration in the tears, a 25-plex Cytokine Panel and the Luminex xMap were used. This platform enables simultaneous measurement of proinflammatory cytokines, Th1/Th2 distinguishing cytokines, nonspecific acting cytokines, and chemokines.

**Results:**

We demonstrated the following: (i) 25 cytokines/chemokines expressed highly variable interactions with buffers and matrices. Several buffers enabled recovery of similar cytokine values (regulated and normal T cell expressed and secreted [RANTES], interleukin [IL]-13, IL-6, IL-8, IL-2R, and granulocyte-macrophage colony-stimulating factor [GM-CSF]); others were highly variable (monocyte chemotactic protein-1 [MCP-1], monokine induced by interferon-gamma [MIG], IL-1β, IL-4, IL-7, and eotaxin). (ii) Various extraction buffers displayed significantly different recovery rates on the same sponge for the same cytokine/chemokine. (iii) The highest recovery rates were obtained with the Merocel ophthalmic sponge except for tumor necrosis factor-α: the Weck-Cel ophthalmic sponge showed the best results, either with cytokine standards loaded onto sponges or with tears collected from the inner canthus of the eye, using the sponge. (iv) IL-5, IL-10, and interferon-α were not detected in any tear sample from four normal human subjects. Twenty-two cytokines/chemokines that we detected were extracted from the Merocel sponge to a satisfactory recovery percentage. The recovery of IL-7 was significantly lower in the extracted Merocel sponge compared to the diluted tear samples. The cytokine/chemokine extraction from tears showed the same pattern of extraction that we observed for extracting the standards.

**Conclusions:**

Simultaneous measurement of various cytokines using ophthalmic sponges yielded diverse results for various cytokines as the level of extraction differs noticeably for certain cytokines. A second set of controls (standard curves “with sponges”) should be used to delineate the extent of extraction for each cytokine to be analyzed. Many cytokines/chemokines were detected in tear samples collected with the Merocel sponge, including many that have been implicated in ocular surface disease. Luminex detection of cytokine/chemokine profiles of tears collected with Merocel sponges and extracted with buffer EX1 may be useful in clinical studies, for example, to assess cytokine profiles evaluation in ocular surface diseases.

## Introduction

The mucosal surfaces are our first immune barriers to the outside world. Thus, these surfaces play a key role in susceptibility to various pathogenic microorganisms. Despite the importance of mucosal immunity, studies of mucosal immunity on conjunctival interface are lacking, making it hard to gain a more comprehensive view of the local immune response. That is largely because analyzing mucosal immune activity requires invasive procedures unlike systemic immune responses, which are easily measured from blood or urine samples. Understanding of the importance of immunological mechanisms underlying the diseases on the conjunctival interface and identification of associated immunological markers have grown enormously over the past ten years as several immunological and ophthalmological studies have investigated the relationships between ocular diseases and immunological parameters [1-4]. These parameters are often involved in complex immunological cascades, and these relationships could be variable; for example, cytokines may have different effects in different cell populations at different times and in the presence of other cytokines [5]. Measuring cytokines/chemokines quantitatively is important, especially in cases when the extent of correlation between different cytokines must be assessed or the balance between levels of cytokine expression must be quantified. With this in mind, cytokine profiling in tears could be useful as a diagnostic or prognostic marker in various ocular surface diseases. Although measuring humoral immune components in blood or urine requires simple and common methods, analyzing immune parameters in tears is still difficult.

Nevertheless, collecting tears, unlike fluids from other mucosal surfaces, is demanding, and obtaining reproducible and unaltered samples is challenging because the recovered tear volumes are small. Several methods are available for tear collection, such as microcapillary glass tubes [6,7], Schirmer strips [8-10] (Preferred Practice Pattern), and the use of sponges, but drawbacks are associated with each approach [11].

Aspiration of tears by glass capillary tubes or pipettes can yield volumes of 20–50 µl, but collecting is tedious, time-consuming, and sometimes uncomfortable for anxious patients and children. Furthermore, by touching the conjunctiva either with capillary tubes or pipettes, it is possible to provoke the production of reflex tears [12,13], which are different in composition compared to basal tears. Overall, this method seems inadequate for incorporation in clinical trials where reproducible data should be collected from large cohorts especially when children are involved.

Schirmer’s test is commonly used in clinics for diagnosing dry eye disease by measuring tear volume. There is an established procedure to recover tears from a Schirmer strip for measuring multiple tear cytokines with Luminex technology [14]. A possible disadvantage of this method comes from the fact that tear reflection is very common with Schirmer’s test due to strong irritation by the strip. With tear reflection, the cytokines can be easily diluted.

To overcome these limitations, some authors have reported successfully collecting tears by using ophthalmic sponges [12,15,16]. However, various sponges and extraction buffers were used, making it difficult to assess the feasibility of the protocols and to compare the results. Ophthalmic sponges are also widely used for collecting non-ocular mucosal secretions, that is, cervical fluids and sputum, to measure antibody or cytokine levels [17].

Researchers have shown for oral and genital tract secretion that immunoglobulin recovery from the sponges is consistent and reliable. However, researchers discovered that some cytokines, unlike immunoglobulins, bind tightly to the sponges, and diffusing cytokines out of the sponges during the extraction procedure can be difficult [18,19].

Novel assays using small volumes, that is, Luminex technology [14,20-22], are beginning to replace older methods that have been a major bottleneck for quantitatively measuring a multitude of intact cytokines in small volumes as tears and could help to expand our understanding of the immunoregulation at the ocular surface. However, the ultimate outcome could be affected by the tear collection method chosen and the consistency of the extraction protocol. Therefore, the aim of our study was to compare interactions between various ophthalmic sponges and extraction buffers, explore their effects on cytokine recovery, and establish an optimized protocol for quantifying many cytokines using the Luminex technology.

## Methods

### In vitro loading of ophthalmic sponges

Sponges: Merocel Sponge points (polyvinyl alcohol, Medtronic Xomed, Inc., Ophthalmics, REF 400115, Abingdon, Oxfordshire, UK), Pro-Ophta lancet sponges (polyvinyl alcohol, Lohmann & Rauscher International GmbH & Co. KG, REF 14917, Rengsdorf, Germany) and Weck-Cell (polyvinyl alcohol, Medtronic Xomed, Inc., Ophthalmics, REF 400115, Abingdon, Oxfordshire, UK) were loaded with 25 cytokine/chemokine standards, granulocyte-macrophage colony-stimulating factor (GM-CSF), interleukin (IL)-1β, IL-1RA, IL-6, IL-8, tumor necrosis factor-α (TNF-α), interferon-γ (INF-γ), IL-2, IL-2R, IL-4, IL-5, IL-10, IFNα, IL-7, IL-12p40/p70, IL-13, IL-15, IL-17, eotaxin, interferon gamma-induced protein 10 (IP-10), monocyte chemotactic protein-1 (MCP-1), macrophage inflammatory protein-1α (MIP-1α), MIP-1β, monokine induced by interferon-gamma (MIG), regulated and normal T cell expressed and secreted [RANTES], from the Human Cytokine 25-Plex Panel Kit (Invitrogen, CatNo. LHC0009 LifeTech Austria, Wien, Austria). The lyophilized standards were reconstituted with the diluent provided with the kit. Each standard was diluted so that 50 μl of the diluted immune marker absorbed by sponges would have a final concentration in the extraction volume equal to the median of the standard curve if 100% of the material was recovered. As a negative control, an additional sponge was loaded with the extraction buffer alone and tested; the resulting value was subtracted from the test values. As a positive control, a volume of the diluted cytokine was added directly to the extraction buffer, and the result was regarded as 100% value. All samples were run in duplicate.

### Extraction of samples from ophthalmic sponges

Each sponge was weighed before and after sample loading to calculate the volume adsorbed onto the sponge. The sponges were then equilibrated in different extraction buffers: EX1 (PBS; 4.3 mM Na_2_HPO_4_, 137 mM NaCl, 2.7 mM KCl, 1.4 mM KH_2_PO_4_, pH 7.4 – supplemented with an additional 0.25M NaCl), EX2 (PBS/0.25 M NaCl/10% FCS, pH 7.4), EX3 (PBS/0.25 M NaCl/0.1% Tween-20, pH 7.4), EX4 (Assay Diluent, provided with Human Cytokine 25-Plex Panel Kit, Invitrogen, LifeTech Austria, Wien, Austria), EX5 (50 mM Tris/0.15 M NaCl/10 mM CaCl_2_, pH 7.6), EX6 (50 mM Tris/0.15 M NaCl/10 mM CaCl_2_/ 0.1% Tween-20, pH 7.6), and EX7 (Tris/0.25 M NaCl, 1% FCS after elution, pH 7.4). All buffers were supplied with aprotinin (0.1 mg/ml; CatNo. A2132.0025, AppliChem, Dresden, Germany). Sponges were equilibrated in 600 μl of EXs for 30 min at 4 °C and centrifuged at 16,000 x g in a Spin-x centrifuge filter unit to separate the extracted samples from the sponge. As for clinical samples analysis, each individual sponge was weighed to determine the volume of secretions absorbed into the sponges. In calculating the final concentration of the immune components measured in the secretions, a dilution factor was determined based on the following formula: dilution factor = [(x - yg) + 0.6g of buffer]/ (x - yg), where x equals the weight of the sponge after collection and y is the weight of the dry spear.

### Tear collection 1

Ten healthy subjects were recruited from the research staff of the Institute of Tropical Medicine and Specific Prophylaxis, the Medical University of Vienna, by advertisement. All subjects were female and comparable in age (33-45 years), occupation and education. The subjects with the history of eye or systemic diseases were not enrolled in this study and for that reason we excluded six subjects. The chosen four female subjects were meeting the following inclusion criteria: no clinical or historical evidence of ocular surface infection, no history of receiving systemic antimicrobials in the preceding 30 days, and no history of receiving topical ophthalmic medications in the preceding 30 days. All participants were willing and able to comply with the study procedures. The tears were collected non-traumatically from the lateral canthus so as to avoid the tear reflex as much as possible, placed separately into the sterile collection tubes, were kept cold during collection, and stored at -80°C until processed.

### Tear collection 2

The ophthalmologist placed a sponge over the lid margin at the junction of the lateral and middle thirds of the lower eyelids and kept the sponge in place for 5 min while the same four healthy female subjects as in tear collection 1 closed their eyes without an anesthetic. The sponges were removed, and tear volume in microliters was recorded. Each sponge was placed into a sterile 2-ml centrifuge tube, centrifuged on 16.000 × *g*, and stored at −80 °C until processed. The study was reviewed and approved by the Ethical Board of the Medical University of Vienna, Austria.

### Multiple×-25 bead array assay

The human cytokine multiple×-25 bead array assay kit for Luminex was purchased from Invitrogen (CatNo. LHC0009). This kit comprises all components necessary for the whole assay procedure to be fulfilled within approximately 6 h hands-on time. The following cytokines were measured: (i) inflammatory panel: GM-CSF, IL-1β, IL-1RA, IL-6, IL-8, and TNF-α; (ii) Th1/Th2 panel: IFN-γ, IL-2, IL-2R, IL-4, IL-5, and IL-10; (iii) cytokine II panel: IFN-α, IL-7, IL-12p40/p70, IL-13, and IL-15, IL-17; (iv) chemokine panel: eotaxin, IP-10, MCP-1, MIP-1α, MIP-1β, MIG, and RANTES. Standard curves for each cytokine (in duplicate) were generated using the reference cytokine concentrations supplied in this kit. To assess possible interference in the assay by the sponges, two standard curves were prepared in parallel: 1) cytokines of known concentrations with the extraction buffers and 2) cytokines of known concentrations loaded onto the sponges with the extraction buffers. The two standard curves were assessed from duplicates consisting of all 25 cytokines/chemokines using a five parameter logistic modeling system. Eight varied dilutions were applied to the standards, and resuspension fluid was used to determine the background. The 25-Plex beads were vortexed and sonicated to disperse aggregates, and washed using a vacuum manifold not exceeding 5 psi. Then, the 25-Plex beads were incubated with 100 µl of standards and the tear samples 1 and 2 for 2 h on an orbital shaker at 500 rpm. Wells were aspirated and washed as through the vacuum manifold. Biotinylated antibodies were added and incubated on the orbital shaker for 1 h with subsequent washes. After streptavidin labeled with R-phycoerythrin was added, the plates were analyzed using a Luminex xMAP instrument (Luminex Technologies, Inc., Austin, TX) to determine the quantities of each protein.

### Analysis

A paired Student *t* test was used for statistical comparison of the samples. Cytokine concentration was determined with the standard procedure given by the manufacturer (Invitrogen, LifeTech Austria, Wien, Austria). The cytokines were adsorbed onto different sponges and extracted with different extraction buffers. The ability of an individual sponge to bind an individual cytokine, independently of the extraction buffer used, was analyzed with an independent Student *t* test. p<0.05 was considered statistically significant.

## Results

### Merocel, Pro-ophta, and Weck-Cel sponges combined with commercial assay diluent as an extraction buffer

**Figure 1:**
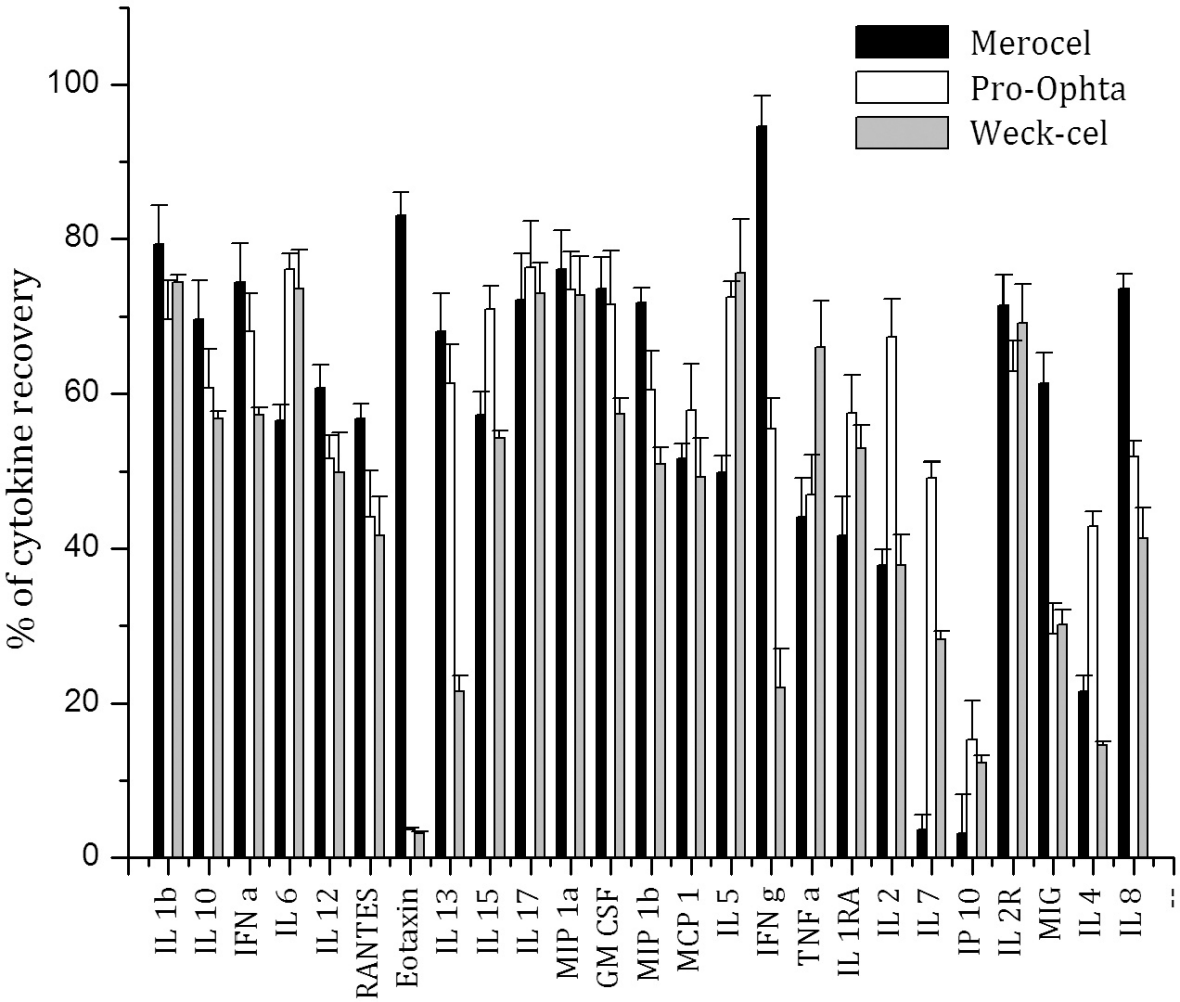
Mean percentages of cytokine/chemokine recovery from Merocel (black columns), Pro-ophta (white columns) and Weck-Cel (gray columns) sponges loaded in vitro with known concentration of 25 cytokines/chemokines and extracted with assay diluent used as an extraction buffer (EX4).

In this study, we used the highly sensitive Luminex technology to quantify cytokines and chemokines adsorbed onto Merocel, Pro-ophta, and Weck-Cel ophthalmic sponges to determine which ophthalmic sponge is the most suitable for tear collection clinical studies. The first extraction buffer used to separate cytokines/chemokines from the sponge matrix was the assay diluent supplied by Invitrogen (EX4). Our preliminary experiment revealed that EX4 was not potent enough to extract all cytokines/chemokines from different sponges’ matrices to an acceptable percentage. Although EX4 showed the best performance with the standards alone, the results with the standards loaded onto sponges revealed distinct differences (Figure 1). Only 3.6% and 3.2% of eotaxin was recovered from the Pro-ophta and Weck-Cel sponges, respectively, and 83% was recovered from the Merocel sponges. Only IL-1β, eotaxin, MIP-1a, IFN-α, IL-17, IL-2R, and IL-8 were nearly quantitatively (>70%) recovered from the Merocel sponges; IL-6, IL-15, IL-17, MIP-1a, GM-CSF, and IL-5 from Pro-ophta, and IL-1β, IL-6, IL-17, MIP-1a, IL-5 from Weck-Cel sponges. The low recovery percentage for cytokines loaded onto the three sponges forced us to further develop the extraction process using various extraction buffers.

### Impact of different extraction buffers on the percentage of cytokine/chemokine recovery

**Figure 2:**
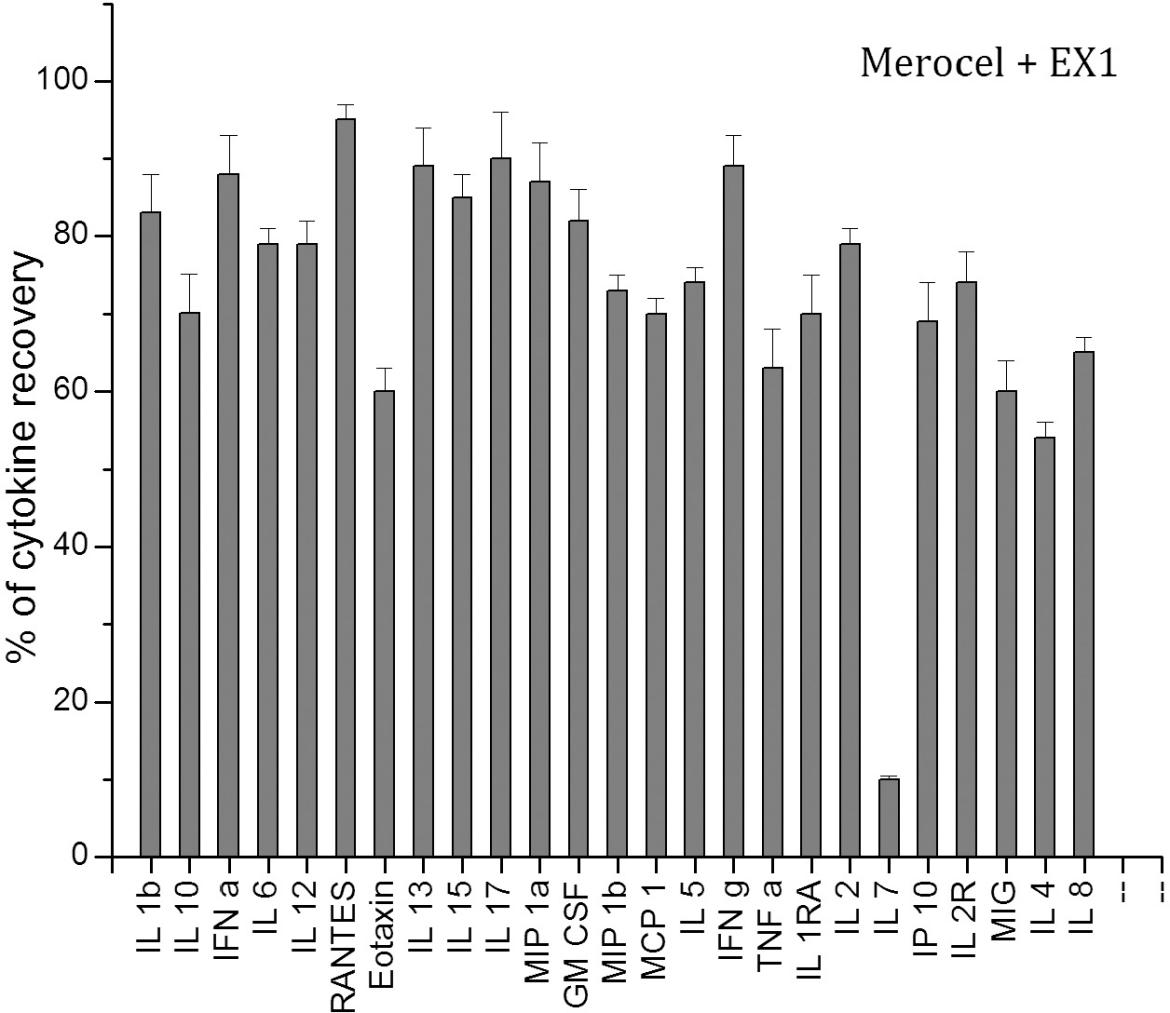
Mean percentages of cytokine/chemokine recovery from the Merocel sponge (gray columns) loaded in vitro with known concentration of 25 cytokines/chemokines and extracted with an extraction buffer 1 (EX1).

The inconsistency in recovery values with all tested extraction buffers was noted. No buffer or sponge recovered all cytokines, although varying combinations gave high cytokine recovery rates. EX1 provided the best percentage of cytokine recovery with the Merocel (p=0.774) and Pro-ophta (p=0.317) sponges, but the absence of cytokine recovery from the Weck-Cel sponges was statistically significant (p<0.001); EX2 and EX3 exhibited good results for extracting the cytokines loaded onto the Merocel sponge (p=0.5088) and the Pro-ophta sponge (p=0.374), respectively. The EX4, EX5, EX6, and EX7 were unable to extract cytokines from all sponges to an acceptable percentage (p<0.005). Several buffers enabled recovery of similar percentages for certain cytokines (RANTES, IL-13, IL-6, IL-8, IL-2R, GM-CSF); others were highly variable (MCP-1, MIG, IL-1 β, IL-4, IL-7, eotaxin). We demonstrated that the extraction buffers consisting of 0.25 M NaCl in PBS were more efficient for extracting the cytokines than the buffers consisting of Tris/0.15 M NaCl/10 mM CaCl_2_ and using Tween-20 had no impact on the extraction (data not shown). Figure 2 depicts the recovery of 25 cytokines and chemokines from the Merocel sponge by using EX1.

### Impact of various sponges on the percentage of cytokine/chemokine recovery

**Figure 3:**
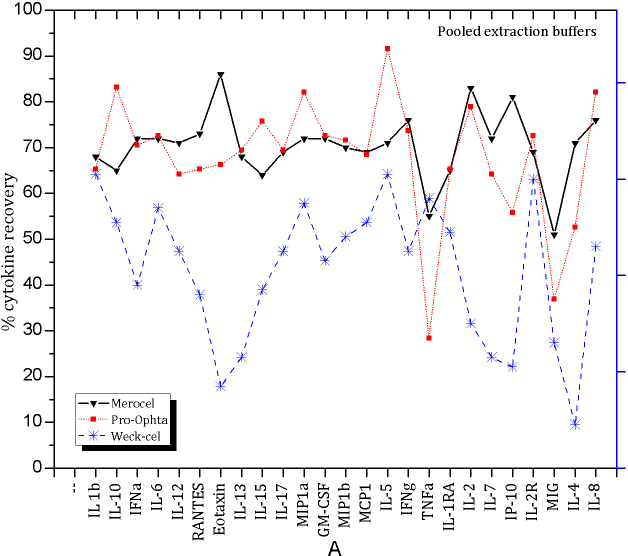
The percentages of cytokine/chemokine recovery from the Merocel (down triangle), Pro-ophta (square) and Weck-Cel sponges (star). Values for the extraction buffers (EX1-EX7) were pooled to visualize the impact of sponge type on the cytokine recovery.

To visualize the effect of the Merocel, Pro-ophta and Weck-Cel sponges on cytokine recovery, we pooled the recovery rates gained with different extraction buffers (Figure 3). The Merocel sponges showed the best recovery rates for all cytokines except TNF-α (p=0. 2) for which the Weck-Cel sponges demonstrated highest percentage recovery values (p=0.25). The Pro-ophta sponges performed not significantly better than Weck-Cel except IFN-α (Pro-ophta: p=0.087, Weck-Cel p=0.0004), IL-2R (Pro-ophta: p=0.597, Weck-Cel: p=0.151), IL-1RA (Pro-ophta, p=0.157, Weck-Cel: p=0.002) and IFN-γ (Pro-ophta: p=0.033, Weck-Cel: p=0.002). The best recovery for TNF-α was obtained with the Weck-Cel sponge (Merocel: p=0.2, Pro-ophta: p=0.0007, Weck-Cel: p=0.25).

### Determination of cytokine/chemokine presence and concentrations in healthy subjects’ tears

Based on these results, we chose the Merocel sponge combined with EX1 buffer (MEX1 protocol) to extract cytokines/chemokines from tears. Twenty-two out of 25 cytokines and chemokines were detected in tears of healthy volunteers. IL-5, IL-10, and IFN-α were not detected in any sample. Table 1 demonstrates the concentrations of the cytokines and chemokines determined using the MEX1 protocol. We have shown that the cytokine/chemokine extraction from tears had the same extraction pattern as we observed for extracting the cytokine standards. The recovery of IL-7 was significantly lower in the extracted sponges compared to the diluted tear samples.

**Table 1:** Concentration (pg/ml) of cytokines/chemokines diluted in assay buffer or extracted with EX1 from Merocel sponges.

Concentration (pg/ml) of cytokines diluted in assay buffer or extracted with EX1 from Merocel sponges		
Cytokines/Chemokines	T	LT
IL-1RA	7723.2±973.0	6710±844
IP-10	4630.9±334.8	4340.9±348.7
IL-8	480.1±32.2	459.8±34.6
MCP-1	101.36±33.9	91.0±8.7
IL-7	133.9±6.8	5.9**±0.1
IL-6	5.7±0.3	4.6±0.2
IL-1β	<0	<0
IL-5	0.34±0.02	0.29±0.02
RANTES	32.5±1.9	27.3±1.6
Eotaxin	22.9±0.9	20.9±1.1
IL-2	24.5±3.2	22.9±1.8
TNFα	4.2±0.5	3.5±0.09
IL-12	27.6±1.2	25±1.1
IL-13	1.1±0.1	0.9±0.08
IL-15	56.7±4.3	53.3±4.3
IFNγ	2.6±0.2	2.3±0.4
IL-10	0.24±0.08	0.2±0.07
GM-CSF	29.4±2.1	22±1.9
IL-4	19.2±1.1	9.2*±0.5
MIP1β	43.7±3.2	30.1±2.2
IFNα	<0	<0
IL-17	76.6±5.8	69.7±5.44
MIP1α	35.0±3.2	27.1±2.2
IL-2R	62.3±5.5	42.8±4.8
MIG	296.6±121.0	270±2.2

Mean values±SD, * p<0.05 versus cytokine diluted in assay buffer and calculated from same buffer standard curve

## Discussion

The purpose of this study was to optimize the protocol for quantifying cytokines from tears using ophthalmic sponges for the Luminex technology. Tear collection with glass capillaries, the most widely used method for taking samples and the best method for avoiding tear reflex, is often impractical for remote area field studies because it is tedious and time-consuming for health workers who cannot collect tears from a large number of patients with this method in one day. We compared three ophthalmic sponges, Merocel, Pro-ophta, and Weck-Cel, combined with seven different extraction buffers. We showed that cytokines/chemokines were recovered from the Merocel sponges more efficiently than from the Pro-ophta sponges, while recovery from the Weck-Cel sponges was lower and more variable. This could be explained by the chemical compositions of the sponges analyzed. The Merocel polyvinyl alcohol sponge material has 100% open pores in a structure with no dead-end pockets that hold residues (Merocel). The Merocel sponges are highly absorbent and fast-wicking. The Pro-ophta sponges are also made from polyvinyl alcohol as Merocel but produced by a different company. Extracting cytokines from the Pro-ophta sponges showed a similar trend as for Merocel. In contrast, the Weck-Cel sponges are made from highly absorbent, natural cellulose material, and maintain rigidity during the wicking process. Weck-Cel sponge materials may have micropockets that can trap starch/sulfate residues. These sponges must be washed with various solutions to remove the residues. Some of the starch residue may be trapped in the polymer structure of the sponge where washing solutions cannot reach. This makes it impossible to remove the residues from the final sponge product and could possibly be applied on cytokine detachment from the sponge [23]. The relatively low recovery of certain cytokines that we observed with all types of used sponges may have a few explanations. It may be the consequence of a physical entrapment of the cytokine within the sponge matrix that is more pronounced with the Weck-Cel sponge than with the Merocel sponge as Merocel is fast-wicking due to the 100% open pores material.

Researchers have hypothesized that IL-4 has characteristic stability problems, suggesting that the molecules are unstable in the extraction buffer [19]. An alteration in the structure of IL-4 may also expose residues in the molecule that react with the polymer matrix of the sponge, resulting in binding and entrapping cytokines within the sponge matrix. In addition, IL-4 possesses three disulfide bonds, and this could influence its recovery from sponge matrix. Researchers have shown that percent recovery rates decrease in correlation as the number of disulfide bonds increases [24]. This hypothesis is supported by our results on IL-7 as we got almost undetectable levels of IL-7 after the extraction no matter which extraction buffer we used.

Our results contrast with the published data reporting on the advantages of cellulose sponges for collecting and analyzing mucosal fluids [25]. Van Agtmaal et al. found no differences in protein recovery rates from tears using cellulose sponges compared to capillary tube usage. Furthermore, Weck-Cel sponges are widely used for cervical fluids and showed satisfactory results for immunoglobulin recovery. Recovering cytokines was more problematic [25], and optimized protocols were consequently published [15,26].

Nevertheless, our results are in line with a study that analyzed the recovery percentage of cytokines from Weck-Cel determined with enzyme-linked immunoabsorbent assay. In this study by Castle et al. [26], the performance of the cellulose-based Weck-Cel sponges was inferior to Merocel as almost no IL-4 and IFN-γ recovery was obtained.

Since cytokines function as chemical messengers for regulating the innate and adaptive immune systems, comprehensive tests for measuring a large set of cytokines to gain a better understanding of the underlying immune regulation are important. The ability to analyze an entire spectrum of cytokines/chemokines in a single sample could give us information relevant to the response on a systematic level. The Luminex strategy may be used to evaluate the cytokine/chemokine content in tear samples in clinical studies, for example, for evaluating cytokine profiles in ocular infections.

We showed that simultaneous measurement of different cytokines using ophthalmic sponges yields different results for different cytokines as the level of extraction differs noticeably for certain cytokines. This is probably due either to the different tertiary sponge structures leading to more physical entrapment or to differences in chemical degradation. However, although in no case 100% of the individual cytokines was recovered from the sponges, this method extracted cytokines from the sponge matrix reproducibly and reliably.

Therefore, we recommend having a second set of controls (standard curves “with sponges”) to delineate the extent of extraction for each cytokine, and these values (and not the values of the cytokine standard alone with the buffer), after the extraction process, should be regarded as 100% reference standard cytokine values. That could be an approach for overcoming a limitation of this technology and the fact that certain cytokines are not recoverable in an acceptable percentage from the sponges.

When using ophthalmic sponges, we recommend using the Merocel sponge as the first choice due to the best recovery rates for almost all cytokines/chemokines. Many cytokines/chemokines were detected in tear samples collected by using the Merocel sponge, including many that have been implicated in ocular surface disease. The main limitation of this sponge is the almost undetectable levels of extracted IL-7 and low levels of IL-4 with any of the extraction buffers used. Extracting IL-4 from all tested sponges is generally problematic and needs further improvement.

Optimizing the extraction process and the choice of an adequate sponge allows us to test cytokine and chemokine concentrations in tears from normal subjects in more detail. We did not detect IL-5, IL-10, and IFNα in any sample. These results are in contrast with the published data reporting the concentration of cytokines in tears from normal subjects [27]. This inconsistency could be explained by the following: 1) in both studies, the population size was limited (n=9 and n=4); 2) this difference may be accounted for by the different Luminex kits used for the concentration determinations. Determining a cytokine concentration in healthy subjects is particularly important as a reference to compare with those of patients with ocular surface diseases. Analyzing differences in cytokine/chemokine levels could give us knowledge to help understand ocular surface cytokine patterns. This could then lead to developing strategies for combating ocular surface diseases.
